# Clinical, radiological, and genetic variation in pontocerebellar hypoplasia disorder and our clinical experience

**DOI:** 10.1186/s13052-022-01349-9

**Published:** 2022-09-08

**Authors:** Serap Bilge, Gülen Gül Mert, Özlem Hergüner, Duygu Özcanyüz, Sevcan Tuğ Bozdoğan, Ömer Kaya, Cengiz Havalı

**Affiliations:** 1 Department of Pediatric Neurology, College of Medicine, Çukurova University, Adana, Turkey; 2 Department of Medical Genetics, College of Medicine, Çukurova University, Adana, Turkey; 3 Department of Radiology, College of Medicine, Çukurova University, Adana, Turkey; 4 Department of Pediatric Neurology, Health Sciences University Bursa High Specialization Training and Research Hospital, Bursa, Turkey

**Keywords:** Pontocerebellar Hypoplasia, Neurodegenerative Disorders, Microcephaly, Epilepsy

## Abstract

Pontocerebellar hypoplasia (PCH) constitutes a heterogeneous neurodegenerative/neurodevelopmental disorder of the pons and cerebellum with onset in the prenatal period. Our study aimed to present different clinical and radiological manifestations of our genetically diagnosed PCH patients.

**Method: **Six patients were enrolled in this study from September 2018 to March 2021. All the clinical radiological and genetic investigations were done at Cukurova University Medical School.

**Results:** Five children were diagnosed genetically and categorized under one of the types of PCH (type 10,7,11). Homozygous mutations in *CLP1* In PCH type 10, *TOE1* in PCH type 7, and *TBC1D2*3 in PCH type 11 were respectively detected. Pateint with PCH type 11 and female patient with PCH type 7 could walk and speak few words. Male patient with PCH type 7 had disorder of sex development.

**Conclusion:** According to our study, PCH is a rare neurodegenerative disease, although some types are static as PCH11 male gender and PCH7 female gender. Some clinical features are specific to a definite type. PCH7 express disorders of sex development most apparent in 46 XY. Some ethnic groups could express distinct subtypes. PCH10 is seen in the Turkish population. Radiological imaging is beneficial in pre-diagnosis; all the patients had different pons and cerebellar hypoplasia degrees. Genetic testing like whole exome sequencing -next-generation sequencing is essential in setting the definite diagnosis and determining the type/subtype of PCH.

## Background

Pontocerebellar Hypoplasia (PCH) is a group of neurodegenerative disorders of the pons, cerebellum, and supratentorial regions of the brain that may vary in heterogeneity. However, there are also a few cases in which pons are spared [1]. Severe microcephaly, global developmental delay, and radiological manifestation such as hypoplasia of the pontine and cerebellum are characteristic features of this disorder. Motor and cognitive impairment are seen in all types [2]. PCH has mostly prenatal onset, and the presentation varies from lethal neonatal subtype to milder forms in which children could survive into adolescence [3]. The neurological manifestation of PCH appertains with the malfunction of the cortex and basal ganglia. Contrary to what would be expected, cerebellar symptoms are not a presenting clinical symptom in any of the PCH types [3–5]. Genes related to PCH play a role in RNA metabolism, protein translation, and sometimes mitochondrial respiratory chain regulation [6]. Defects in the associated genes were based on while proposing PCH classification. In 1993, the first classification of PCH included two subtypes, PCH1, and PCH2. Since the first original description of PCH, the phenotype has been profoundly broadened. Many different subtypes were added to the classification of PCH which was initially based upon distinct clinical, radiological, or biochemical features (like optic atrophy, and CSF lactate elevation), and later followed by the finding of associated gene defects. At least 21 PCH-related genes are listed in the database of OMİM. Currently, there are 15 types of PCH.

PCH1 is characterized by PCH with in addition bulbar and spinal motor neurodegeneration identical to spinal muscular atrophy. Early reports describe PCH1 as a neonatally lethal disorder with polyhydramnios, congenital contractures, respiratory failure, and severe muscle hypotonia. Later studies describe sparing of the ventral pons and survival into puberty, thereby broadening the clinical and neuroradiological spectrum of PCH1. Currently, four genes are associated with PCH1 (PCH1A-D).

PCH2 Probably PCH2A is the most prevalent and best characterized of all PCH subtypes. PCH2A is caused by a homozygous mutation in the *TSEN54* gene. Clinically, PCH2A is distinguished by generalized clonus and incoordination of sucking and swallowing in the neonate. The toddler and young child suffer from spasticity, dystonia/chorea, and epilepsy and show a lack of voluntary motor development. In PCH2A sleeping disorders, recurrent infections, apneas, and problems in temperature regulation are reported in the majority of patients. Microcephaly, usually absent in the neonatal period, is progressive, and caused by supratentorial atrophy. ‘dragonfly’ configuration of the cerebellum on brain MRI, resulting from severely affected hemispheres and relative sparing of the vermis is very characteristic in this type. There are many subtypes in this group These subtypes are classified as PCH2A PCH2B, PCH2C, PCH2D, and PCH2F.

PCH3 is characterized by pontocerebellar atrophy, thin corpus callosum, progressive microcephaly, seizures, small stature, facial dysmorphism, and in some patients optic nerve atrophy. The extrapyramidal movement disorders that are typically seen in PCH2 are absent. mutations in the *PCLO* gene are the cause of this disorder.

PCH4&5 Clinically, PCH4 presents as a severe form of PCH2, with prenatal onset of symptoms including polyhydramnios and congenital contractures, prolonged neonatal clonus, hypertonia, and primary hypoventilation requiring prolonged mechanical ventilation. Survival beyond the neonatal period is rare. mutation in *TSEN54* is blamed.

PCH6 phenotype consists of severe early onset epilepsy, progressive global atrophy including pons and cerebellum, lactic acidosis, and/or mitochondrial respiratory chain defects PCH6 is caused by mutations in the nuclear-encoded mitochondrial Arginine tRNA-synthetase (*RARS2*).

PCH7 is characterized by the rare combination of PCH with disorders of sex development. Patients show a severe developmental delay, profound truncal hypotonia with hypertonic limbs and brisk deep tendon reflexes, and seizures. Disorders of sex development. mutations in *TOE1* are blamed.

PCH8 microcephaly, severe developmental delay (although some patients were able to walk independently), dystonic posturing, and/or choreiform movements are the main characteristic features of the type. Some patients had (congenital) contractures and seizures. PCH8 might be considered a 'non-degenerative’ form of PCH and is caused by mutations in the *CHMP1A* gene.

PCH9 is characterized by progressive microcephaly, profound neurodevelopmental delay, and cortical visual impairment. Facial dysmorphisms are reported with dental abnormalities in a minority of patients. The presence of axonal neuropathy is reported in older patients and is probably age-dependent PCH9 caused by mutations in the *AMPD2* gene. Radiologically there is Figure of eight brainstem. PCH10 Some families of Turkish origin have been reported with PCH10. Mutations in *CLP1* have been identified as the causal gene defect.

PCH11 Families with PCH11 have been reported. Patients were homozygous for truncating or splice site mutations in the *TBC1D23* gene. It is considered to be non-degenerative form of PCH autistic features, attention deficit-hyperactivity, independent walking, ataxia, developmental delay are the main features. PCH12 is caused by homozygous or compound heterozygous mutation in the *COASY g*ene. PCH13 is caused by homozygous or compound heterozygous mutation in the *VPS51* gene. PCH14 is caused by homozygous or compound heterozygous mutation in the *PPIL1* gene. PCH15 is caused by homozygous mutation in the *CDC40* gene and is a severe autosomal recessive neurodevelopmental disorder characterized by the congenital onset of progressive microcephaly and poor or absent psychomotor development with severely impaired intellectual development apparent from birth. Other features may include spastic quadriplegia, early-onset seizures, chronic anemia, and thrombocytopenia [5].

Lots of problems can be seen in PCH, sleep apnea, feeding problems, epilepsy, movement disorders, rhabdomyolysis, and strongly elevated serum creatinine kinase, especially during infection episodes [2]. There is no definite treatment for any type of PCH, and management is supportive in all types and subtypes [7–11]. PCH9 has been considered to be a potentially treatable disorder because the administration of a purine nucleotide precursor (AICAr) could rescue the phenotype at a cellular level. Follow-up experiments are needed [5].

## Material & methods

Patients who were admitted to Çukurova University Pediatric Neurology Clinic between September 2018 to September 2021 with delay in milestones in more than two developmental domains, microcephaly (occipitofrontal circumference of *Z* scores > –3) and cerebellar volume loss and pons hypoplasia on MRI were retrospectively evaluated, and six of these patients with a genetically established diagnosis of PCH were included in this study. Written informed consent was obtained from the parents of all children. All the patients with a history of TORCH infection, severe preterm birth, a history suggestive of neonatal encephalopathy secondary to perinatal asphyxia, meningitis, or intracranial bleed, and metabolic disease were excluded from the study. Children with associated malformations of cortical development on neuroimaging were also excluded from the study. Associated problems such as epilepsy, optic atrophy, polyneuropathy, and abnormal sex development were investigated and recorded. All the radiological and genetic analyses whole exome sequencing—Next-generation sequencing (Illumina, California, USA) were conducted at Çukurova University. The mean average follow-up was at least three years.

### Statistical analysis

All analyses were performed using IBM SPSS Statistics Version 2010 statistical software package. Categorical variables were expressed in numbers, whereas continuous variables were summarized as mean and standard deviation, and median and minimum–maximum were appropriate IBM Corp. Released 2011. IBM SPSS Statistics for Windows. Version 20.0. Armonk. NY: IBM Corp.

## Results

Six cases were enrolled in the study; five children were diagnosed genetically and categorized under one of the types of PCH (type 10,7,11). The mutation was detected by solo based whole-exome sequencing. Homozygous mutation in *CLP1* c.419G > A(p.R140H) in PCH type 10, *TOE1* c.572A > G(p.N191S) in PCH type 7, and *TBC1D23 (* c.1263 + 1G > A) in PCH type 11 were respectively detected. The variant mutated genes was searched in the parents. The Parent were found to be carriers for the variant mutated genes.

The genetic mutations in patient 1,2,3,4 were known variants and previously reported in the literature and they showed almost the same clinical features, while the homozygous genetic mutation in patient 5 (PCH11) in *TBC1D23 gene* (NM_001199198) Variant: (c.1263 + 1G > A) was novel variant and not previously reported in the literature (Human Gene Mutation Database HGMD).

The inheritance pattern was autosomal recessive. In-silico Parameters: (Deleterious Annotation of genetic variants using Neural Networks)DANN:0.995, Minor Allele Frequency MAF: Previously undetected variant: This variant was identified by whole exome sequence, and both parents of the patients were found to be carriers.. According to American College of Medical Genetics and Genomics(ACMG), the clinical significance of this variant was evaluated as a pathogenic variant.

**Fig. 1:**
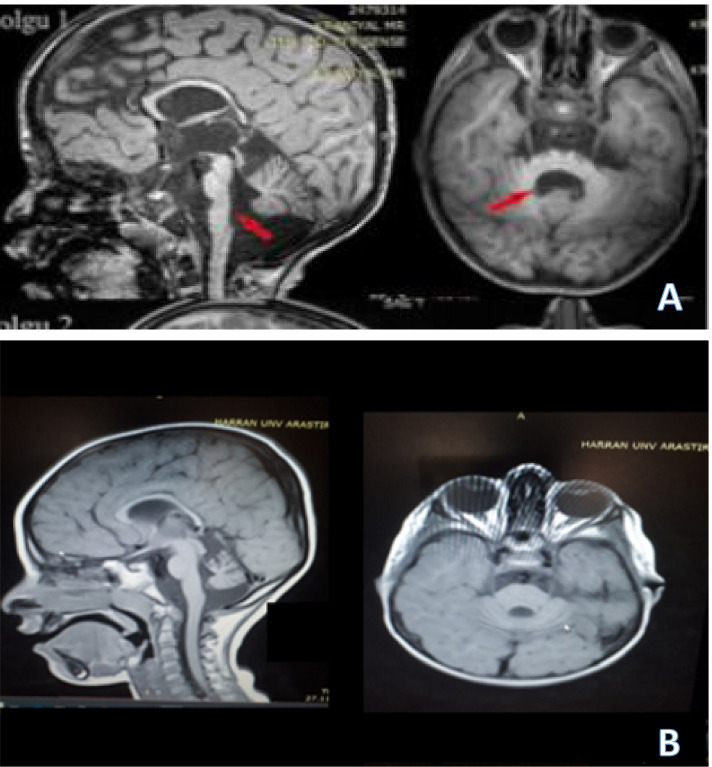
**A** 8 years old boy with PCH type 11, brain MRI ( sagittal and axial sequences) is showing, MRI-Ventral pons flattening, vermis hypoplasia ( blue arrow). Minimal hypoplasis of the hemispheres. **B** 7 years old boy with PCH type 7 brain MRI( sagittal and axial sequences) is showing Vermis hypoplasia. hypoplastic hemispheres, corpıs Callosum hypoplasia, and cortical atrophy

The mean age of the patients that were genetically diagnosed and categorized under one of the types of PCH was 7.3 ± 3.13, at the time of application to pediatric neurology outpatient clinics was 5.05 ± 3.16, and at the time of diagnosis was 5.95 ± 2.96. Three of these patients were male, and two were female. The demographic information, co-morbidities, and radiological and genetic results are shown in Table 1. The radiological manifestation of our two PCH( type 11,7) diagnosed patients is shown in Fig. 1.

**Table 1:** Detailed milestone, demographic, clinical, radiological, and genetic features of the patients

**Pts/families**	**Patient 1/** **Family 1**	P**atient 2** **Fmaily 1**	**Patient 3** **Family 2**	**Patient 4** **Family 2**	**Patient 5** **Family 3**
**PCH Type**	10	10	7	7	11
**Age(years)**	5 (Ex age 5)	4.25	8.75	6.5	12
**Age at diagnosis(years)**	4.5	3.25	6.75	4.5	10.75
**Age at application(years)**	3.5	2.25	5.75	3.5	10.25
**Consanguinity**	Yes	Yes	Yes	Yes	Yes
**HC(cm)**	43	44	47	47	47
**Gender**	M	F	M	F	M
**Birth Weight (Kg)**	Term/2.100	Term/2.200	Term/2.700	Term/3.100	Term/3.200
**Fetal Distress**	No	No	No	No	No
**Birth HC (cm)**	33	33,5	32	33	33
**Head Control (Months)**	7	4	8	5	5
**Sitting Age (months)**	No Sitting	With hand support at 48	18	12	12
**Walking Age (year)**	No walking	No walking	No walking	At 3 Still walking	At 4 Still walking
Standford-Binnet Test	Severe delay	Severe delay	moderate delay	Mild delay	Mild delay
Epilepsy	Yes	No	Yes	No	Yes
Anti-seizure medication	Levetiracetam Vigabatrin Topiramate Phenobarbital	-	Valproic acid Oxycarbamezepine Phneobarbital	-	Levetiracetam
Neuropathy	Yes	No	No	No	No
Pyramidal/ Extrapyramidal symptoms	Yes	Yes	Yes	Yes	Yes
Optic Atrophy	Yes	Yes	No	No	No
Scoliosis/ contractures	No	No	No	No	No
Abnormal genitalia	No	No	Yes	No	No
Genetic	*CLP*1 c.419G > A(p.R140H) Homozygous mutation *reported before	*CLP1* c.419G > A(p.R140H) Homozygous Mutation *reported before	*TOE 1* c.572A > G(p.N191S) Homozygous mutation *reported before	*TOE1* c.572A > G(p.N191S) Homozygous Mutation * reported before	*TBC1D23* (c.1263 + 1G > A) Homozygous mutation novel variant
MRI-Ventral pons flattening	Yes	Yes /Min	No	No	Yes
MRI- Vermis hypoplasia	Yes	Yes/Min	Yes/Min	Yes/Min	Yes
MRI-Hypoplastic hemispheres	Yes	No	Yes	Yes	Yes/Min
Corpıs Callosum Hypoplasia	Minimal	No	Yes	Yes	No
MRI-Cortical atrophy	Yes	No	Yes	Yes	No
MRI-Abnormal myelination	Yes	No	No	No	No
MRI at Birth or specific age	Not available	Normal (at two years old)	Not available	Not available	Not available

*was designed to pay attention to this part

**Fig. 2:**
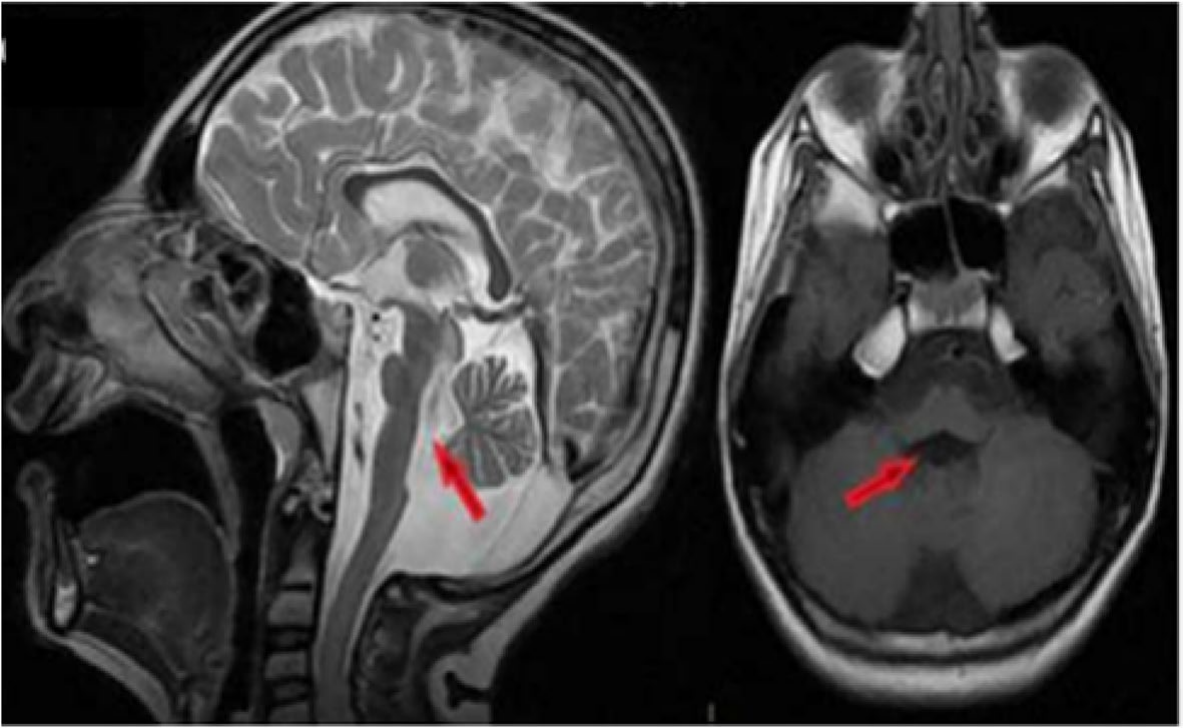
Six years old boy, brain MRI ( sagittal and axial sequences) is showing lateral enlargement of the ventricles and thinning of the corpus callosum and hypoplasia of the serebellum and pons (red arrow)

Besides these (genetically diagnosed and categorized under one type of PCH) patients, a six-year-old male patient with growth retardation, motor, and cognitive development delay applied to our outpatient clinics. He started walking with small steps at the age of four, can speak a few words, and has chew and swallowing disorders. Dysmorphic facial appearance on physical examination was noticed. He had poor social interaction and attended mainstream public school, but he was not doing well and was provided with special education support. Metabolic tests were normal. Hypoplasia of the pons and cerebellum vermis, lateral enlargement of the ventricles, and thinning of the corpus callosum were detected on brain MRI (Fig. 2). Genetic test whole-exome sequencing-NGS was conducted and compound heterozygous mutation in the *BRF1* gene was identified. There are a few cases described in the literature, and this gene mutation was associated with cerebellofaciodental syndrome. Our patient's current findings were found to be compatible with this syndrome.

## Discussion

PCH is an umbrella term that includes a group of heterogeneous, rare, and devastating neurodegenerative disorders. The new developments in genetic testing led to the description of new genes and phenotypes of PCH. So far, 15 types of PCH and 21 genes related to PCH are prescribed [1, 12, 13].

The updated classification highlights two significant aspects of the PCH. Although this disorder is degenerative, PCH8 and PCH11 are both non-degenerative ones [1, 14–16]. Our results are partially in line with other studies because patient 4 (Female gender -PCH type 7) and patient 5 (Male gender-PCH type 11) could walk and speak few words compared to other patients. The studies showed all the types have prenatal onset except PCH2D, PCH2E, and PCH10, which have a postnatal onset. In our study, patients 1,2,4,5 had normal head circumference at birth. These results partly match the results of other studies. At the same time, Laugwitz et al. studied PCH type 11. He identified 18 different clinical subtypes of PCH associated with pathogenic variants in 19 different genes have been described, predicting a loss of protein function. In the study, brain morphometry revealed a pattern of the pontine, brain stem, and supratentorial volume loss similar to PCH2 patients, although less pronounced. Intriguingly, cerebral MRI findings at the age of 1 and 15 years clearly showed progressive atrophy of the cerebellum, especially the hemispheres. In four of the cases reported in the literature, cerebellar hemispheres could be evaluated on the MRIs displayed, and they also showed atrophic foliage. While pontine hypoplasia and pronounced microcephaly are in line with previous reports on PCH11, the observations of this study for postnatal atrophy of the cerebellum argues for a different pathomechanism than in the other forms of PCH. It supports the hypothesis that TBC1D23 deficiency predominantly interferes with postnatal rather than with prenatal cerebellar development [17]. In our study, PCH type 11-.

Patient 5 had only one MRI, so the comparison was not possible whether there was progressive atrophy of the cerebellum, but our patient could walk, speak, and attend school in spite that he was not doing well at school, so we think that the genetic defect determine the timing of cerebellar pathology in all types, but lots of studies should be performed in this field. The updated classification also highlights that PCH type 2 is the most common form of this autosomal recessive disorder; despite this fact, non of our patients experienced this form [1, 2].

In most cases, the disease is uniformly fatal in early life. Life span has ranged from death in the perinatal period to about 20–25 years of age. Only a few individuals-usually patients with PCH type 2-have survived to the second and third decades of life [1, 4]. In our study PCH, type10 male gender died at the age of 5 due to recurrent respiratory infection and aspirations. In contrast, the rest of the patients are still alive, experiencing some problems like movement disorders and epilepsy. The mean age of the our patients on clinic application was 4.0 ± 2.45, and the diagnosis means age was 5.0 ± 2, which seems high. This could be due to the recently decreased cost of genetic testing and recently increased access to massively parallel or next-generation DNA sequencing.

Lots of problems can be experienced in PCH, sleep apnea, feeding problems, and epilepsy. Patients 1, 3, and 5 had seizures and used anti seizures medication. In our study, the expression of the PCH seemed to be milder in females than males. The female patient with PCH type 10 could sit with hand support, and she didn't have any seizures, while the male patients with PCH type 10 couldn't sit and were diagnosed with epilepsy and used anti seizures medication. The female with PCH type 7 didn't have any anomaly in sex development and wasn't diagnosed with epilepsy. In contrast, the male patients with PCH type7 had a disorder with sex development (absence of testis and micropenis was noticed) and he was diagnosed with epilepsy. Ethnicity also could play a significant role in the variation of expression in this disorder. So clinical manifestations could differ from one population to another. Thus lots of studies are needed [1, 2].

PCH does not always mean that there is a genetic background behind it. There are also non-genetic acquired reasons such as congenital cytomegalovirus infections, hemorrhage, ischemia, exposures to teratogenic drugs like phenytoin and valproic acid, and extreme prematurity (< 32 weeks). Other genetic diseases as congenital disorders of glycosylation (type1a), dandy walker syndrome, α-dystroglycan related dystrophies (Walker Warburg, muscle eye brain disease, Fukuyama congenital muscular dystrophies), lissencephaly with cerebellar hypoplasia, *CASK* gene defect, RELN &VLDLR mutations, X-linked hoyeraal-Hreidrasson syndrome, pediatric-onset of spinocerebellar ataxia could mimic PCH and have to be checked especially in unresolved cases (the ones that don't have a genetic diagnosis and are not categorized under one of of PCH types) [18–26]. But it is not understandable why some genetics, such as the *BRF1* gene, are not considered and categorized as one of the PCH genes because PCH type 3 in addition to pontocerebellar hypoplasia they express some facial dysmorphism and dental anomaly and other types of PCH as type 7 could express other problems as disorder of sex develeopment in males. In the future we wonder if other genes, specially *BRF1*, would be included under one the types of PCH.

The shared clinical profiles of 169 PCH patients published by Namavar *et.* were severe microcephaly, seizures, pyramidal/extrapyramidal involvement, and poor psychomotor development. The common clinical profiles of our patients included microcephaly, poor cognition, psychdevelopmental delay, pyramidal and extrapyramidal movements in all patients, and epilepsy in some of them. All the children in our study group were born term, unlike Namavar et al*. study*. Where prematurity was seen in 24% of children with PCH. Our results partially matched Namavar et al*. study* [3] but matched wafik et al. results that presented two cases of PCH10 in the Turkish population. The main complaints of these two cases were severe psychomotor delay, progressive microcephaly, and constipation [27]. However, intrafamilial phenotypic variability was suggested due to the variability in their brain abnormalities and clinical features. At the same time, non of our patients had fetal distress after birth. Neuropathy was seen in our two patients of PCH types 10, which was in line with other studies that showed that neuropathy was mainly seen in PCH 9 and 10, and seizures were seen in patients 1,3, and 5.

Ethnic background is essential in setting some subtypes of PCH since some form is seen in specific ethnicity. PCH10 has only been prescribed in children of Turkish origin, while PCH2E is of Moroccan Jewish origin. Our study is compatible with these results because 2 of our patients were diagnosed with PCH type 10. Each type/subtype of PCH has characteristic features such as disorders of sex development most apparent in 46 XY, which could only see in PCH7. Our study was compatible with these results because the male patient with PCH type 7 had a disorder in sex development while the female one didn’t have such a problem [1, 2].

Radiological imaging helps to specify types/subtypes of PCH in which the pontine, cerebellum, and supratentorial regions of the brain could be affected. Similar radiological findings were observed in patients with identical mutations, but a correlation with clinical severity was not reported. The eighth pattern was seen in patients with PCH type 9 and dragonflies in PCH type 2. In our study, The main feature of MRI is a different degree of cerebellar hypoplasia and pontine involvement; this was compatible with other studies' results. Genetic testing as NGS is generally helping the whole time, but no causative genes could be seen in 40% of PCH cases. [28–30].

There is no specific treatment for any PCH; supportive measures such as gavage PEG feeding in case of feeding problems and sleep monitoring for sleep apnea are required. Anti-seizure drugs such as phenobarbital and topiramate are reported to be very effective in the treatment of seizures in PCH, especially in PCH2A [1, 2]. Our study showed that phenobarbital is effective in the treatment as monotherapy and even in polytherapy. When phenobarbıtal was added to the polutherapy treatment, the seizures were stopped but lots of studies are needed.

### Limitation of the study

PCH is a sporadic disorder; the inability to include all the types is considered a limitation. Informative coronal views are lacking; this seems to be the second limitation.

## Conclusion

PCH is a rare neurodegenerative disease, although some types are static (PCH7 Female gender, PCH11). Some clinical features are specific to a definite type (PCH7 express disorders of sex development most apparent in 46 XY). Some ethnic groups could express definite subtypes (PCH 10 are seen mainly in the Turkish population) but lots of studies are needed. Radiological imaging is beneficial in pre-diagnosis, but genetic testing (generally whole-exome sequencing) is essential in setting the definite diagnosis and determining the type/subtype of PCH.

## Data Availability

At Ass Prof. GGM repository. The datasets used and/or analyzed during the current study are available from the corresponding author on reasonable request.

## Abbreviation

PCH: Pontocerebellar hypoplasia
